# Rapidly progressive COVID-19 viral pneumonia: a report of two patients with a focus on imaging findings

**DOI:** 10.1186/s43055-020-00225-y

**Published:** 2020-06-16

**Authors:** Armin Zarrintan, Reza Javadrashid, Mohammad Kazem Tarzamni, Anita Zarrintan, Masih Falahatian, Mohammad Mirza-Aghazadeh-Attari

**Affiliations:** 1 Department of Radiology, Tabriz University of Medical Sciences, Tabriz, Iran; 2 Medical Radiation Sciences Research Group, Faculty of Medicine, Tabriz University of Medical Sciences, Tabriz, Iran; 3 Department of Internal Medicine, Faculty of Medicine, Tabriz University of Medical Sciences, Tabriz, Iran; 4 Aging Research Center, Tabriz University of Medical Sciences, Tabriz, Iran

**Keywords:** COVID-19, Novel coronavirus, Radiology, CT scan

## Abstract

**Background:**

The novel coronavirus causes viral pneumonia characterized by lower respiratory tract symptoms and 19severe inflammatory response syndrome. Studies have suggested that the virus has a clinical course with the stepwise progression of clinical signs and symptoms and radiologic alterations.

**Case presentation:**

In the present case report, we discuss two patients who presented with mild symptoms and CT imaging not suggestive of COVID-19, but subsequently had a rapid deterioration, with severe involvement happening in CT imaging. One of the patients survived the initial deterioration, but the other passed away.

**Conclusion:**

We suggest that the clinical course of the virus may be rapidly progressive in some patients, and special attention should be paid to patients being treated for the virus outside of the hospital as an outpatient.

## Background

Severe acute respiratory syndrome–coronavirus–2 was the latest pandemic which started from Wuhan, China. This viral disease is characterized by pulmonary involvement, commonly manifested by coughs, rigor, and fever. Diagnosis of the disease is done by molecular assays, but CT imaging has also been proven detrimental in timely diagnosis and has shown to have an acceptable sensitivity [1]. The disease causes bilateral, multifocal ground-glass opacities in the lungs. The clinical course of the disease has been studied, and it has been revealed that CT findings may be useful in determining the progression of the disease and the clinical stage of the disease. These studies are done on a limited number of patients, and most of them report information gathered from cases originating from mainland China [2].

Here, we report two patients who had normal CT findings before being hospitalized but harbored symptoms suggestive of COVID-19.

## Case presentation

### Case one

**Fig. 1:**
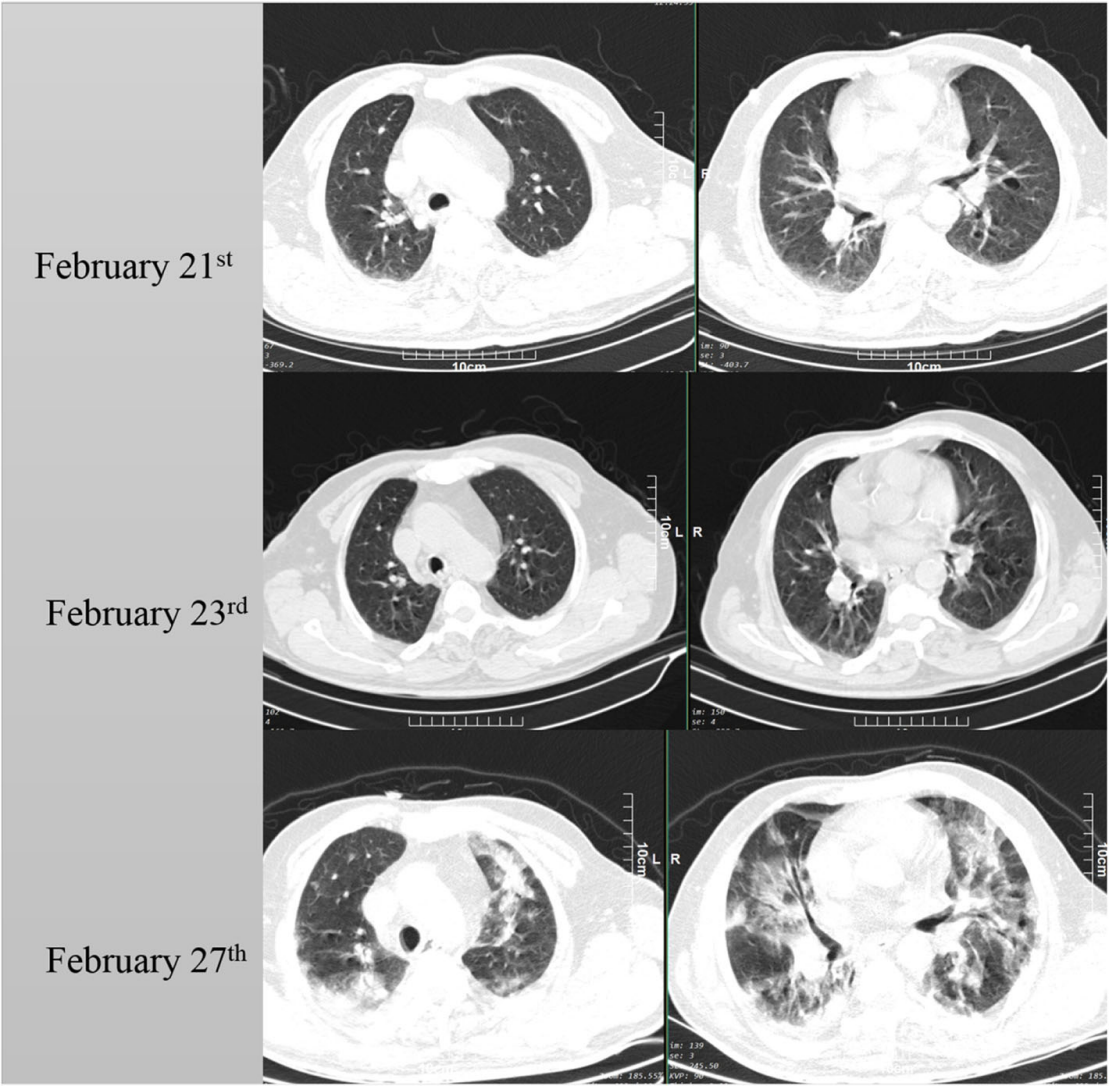
The first and second rows show the chest CT scan which was obtained at the emergency department, which shows no significant findings consistent with COVID19 except some linear atelectasis. The third row shows the chest CT on February 27 which has multifocal bilateral ground-glass opacities with some airspace consolidation suggestive of COVID19

A 63-year-old male patient presented on February 21 to the emergency department. The patient complained of fever, cough, and myalgia. The patient had diabetes mellitus from 5 years ago, with the diabetes being treated by an endocrinologist, and was otherwise healthy and had an active lifestyle. The patient reported symptoms starting from 3 days earlier. Respiratory rate was 17, pulse rate was 93, fever was 38 °C, and blood oxygen saturation was 95% in ambient air. A chest CT scan was obtained from the patient which showed nonspecific findings (few linear and subsegmental atelectasis at lower lobes) which were not suggestive of COVID-19. The patient was put under supervision, and the molecular assay was done to determine SARS-CoV-2 infection. The results were negative, and the patient had infectious disease consultations done. The patient did not have the necessary criteria for hospitalization based on guidelines issued by the ministry of health, which were adapted from those of the center of disease control of the USA and world health organization ((https://irimc.org/Portals/0/NewsAttachment/%20%20%20%20%20%20%20.pdf) [3, 4]). The patient presented to the emergency department on February 23 with fever and exacerbation of dyspnea and complained of symptoms, coupled with headache, nausea, and lack of appetite. The patient had a molecular assay done to rule out COVID-19, which came positive for the virus. The patient had a second CT scan, which showed no significant findings similar to the previous one. The patient was hospitalized for further evaluation regarding his fever at the internal medicine ward. On February 27, the patient had progressive dyspnea, with the continuation of fever and other mentioned symptoms. Blood oxygen saturation was measured to be 81 percent. The patient had a third CT scan, which showed bilateral diffuse ground-glass opacities and airspace consolidation suggestive for COVID-19 (Fig. 1). The patient was admitted to the intensive care unit after consultation from internists and infectious disease specialists. The patient was put on empiric antibiotic therapy and lopinavir ritonavir combined therapy. The patient had decreasing oxygen saturation levels, amide oxygen therapy using a mask with a reservoir bag. The patient was intubated on March 2 and passed away on March 3. Lab data of the patient are summarized in Table 1.

**Table 1:** Lab data of the patients being studied

	Patient 1 tests on February 23^r^	Patient 1 tests on February 27	Patient 2 tests on march 26	Patient 2 tests on march 29
RBC (10^6^ /mm^3^)	4.85	3.42	5.36	4.43
Hb (gr/dl)	14.7	10.4	14.5	13.3
Platelets (1000/mm3)	174	181	129	487
WBC (lymph) (1000/mm^3^)	7.2 (3.6)	5.3 (0.5)	8.6 (2.27)	10.9 (2.61)
Urea (mg/dl)	50	95	25	23
Creatinine (mg/dl)	1.6	1.85	0.77	0.7
AST (IU/L)	124	174	42	45
ALT (IU/L)	97	107	32	30
LDH (IU/L)	819	864	734	416
CPK (IU/L)	N/A	875	73	67
CRP (Quantitative)	Positive 2	Positive 4	Positive 3	Positive 2
ESR (mm per hour)	N/A	121	104	87

### Case two

**Fig. 2:**
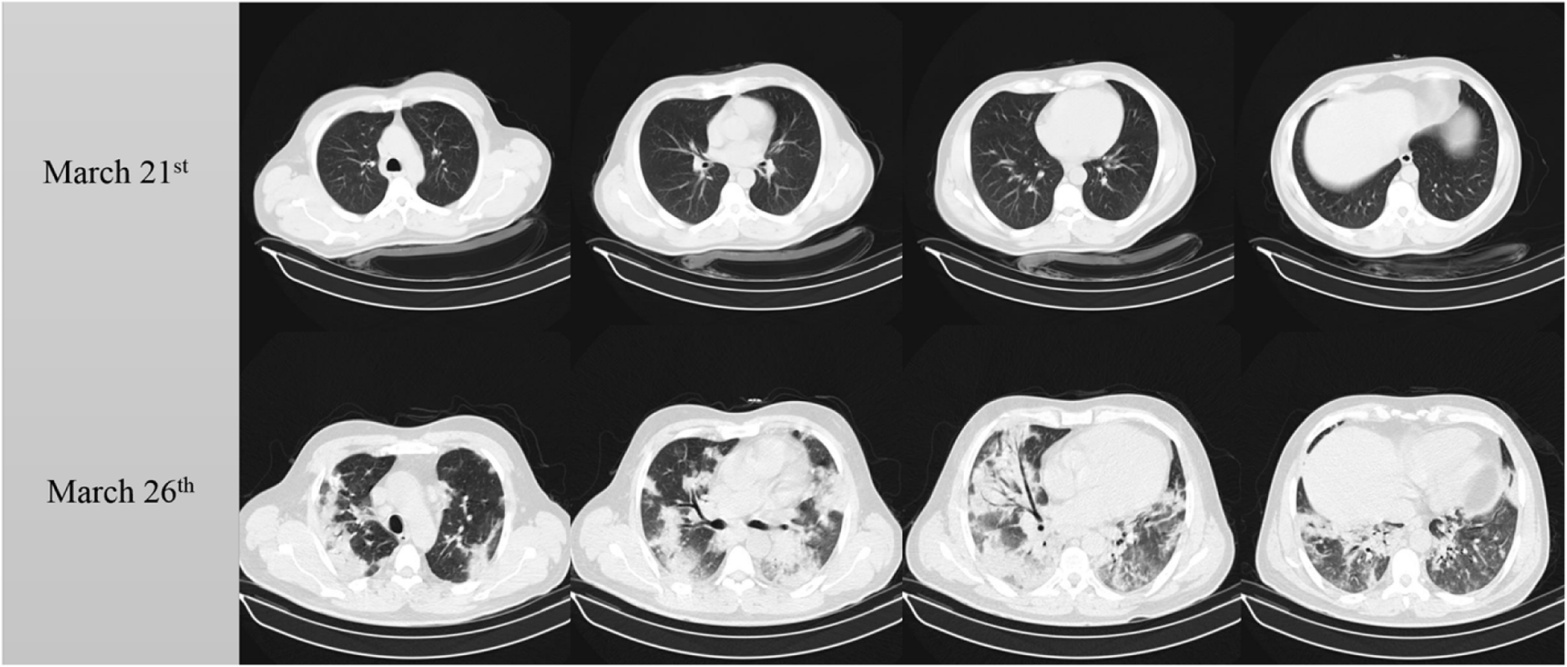
The first row is the chest CT which is obtained at the first presentation of the patient to the emergency ward, and the second row is the chest CT which is obtained days after. As it is obvious, the first CT scan shows the normal appearance, and the following one shows multiple bilateral ground-glass opacities with airspace consolidation and some degree of bronchiectasis in the lower lobes

A 52-year-old male with no pre-existing condition presented on March 21 to the emergency ward with coughs and mild myalgia from 4 days ago. The patient had a respiratory rate of 14, a pulse rate of 76, a body temperature of 36.7 °C, and a blood pressure of 120/65. The patient had contact with a COVID-19 positive case and was thus evaluated for the virus. The molecular assay revealed the patient to be infected. The patient also had a CT scan, which showed no significant finding. The patient was advised to self-quarantine in-home and did not receive any medication. The patient again presented to the emergency department on March 26, this time, complaining of progressive dyspnea, fever, worsening coughs, and loss of appetite. The patient had a second CT scan, which showed bilateral and diffuse multifocal ground-glass opacities and airspace consolidation with some degree of bronchiectasis in the lower lobes (Fig. 2). The patient was hospitalized in the infectious disease ward and was treated with empiric antibiotics, hydroxychloroquine, and oseltamivir. The patient was discharged from the hospital on April 1. Lab data of the patient are summarized in Table 1.

## Discussion

Since the emergence of the novel coronavirus, much attention has been given to its clinical course and clinical manifestations such as the previous coronavirus pandemics [5]. Early studies have suggested that the disease has a course depending on clinical outcome and from the start of initial symptoms to discharge from hospital or death, a 19–21-day interval exists. These studies have also suggested that patients experience sepsis, ARDS, acute cardiac injury, acute kidney injury, and secondary infections before death [6]. Parallel to this, scholars have tried to suggest a radiologic staging based on disease transmission. A study conducted by Bernheim et al. classified patients into three groups based on CT imaging: Group one, those with CT imaging taken on days 0–2 from initiation of symptoms, group two, those with images taken on days 3–5, and the third group on days 6–12. As the duration between CT imaging and initiation of symptoms increased, the frequency of imaging findings and severity of lung involvement also increased. The mean total severity score was 1, 4, and 6 for groups one, two, and three, respectively [7]. In our patients, lung involvement increased substantially in a matter of days (4 days in case one and 5 days in case two). Our findings suggest that the proposed staging can be useful in the general population, but certain patients may experience swift deterioration, both in clinical condition and radiologic findings, as our patients also had a rapid progression of clinical signs and symptoms alongside radiologic progression. Another staging system was proposed by Jin et al. This staging was based on specific radiologic findings occurring in the course of the disease. The staging consisted of 5 stages, with consolidation appearing in the second week after symptom initiation. The occurrence of consolidation was also in the second week in our patients, but early imaging findings in the first week did not occur based on the proposed timeline [8]. Furthermore, these two patients showed a rapid deterioration not previously mentioned in clinical studies [9].

## Conclusion

In conclusion, the clinical course of COVID-19 may not follow that of which is presented in available scientific literature, and more attention should be paid to patients not being treated in hospitals, as they can present severe symptoms without any prior radiologic signs.

## Data Availability

All data and materials are available based on reasonable request, according to the guidelines of the institution in which the study was approved, and based on the guidelines regarding sharing patient information.

## Abbreviations

COVID-19: Coronavirus disease 2019
SARS-Cov-2: Severe acute respiratory syndrome–coronavirus–2
CT: Computed tomography
ARDS: Acute respiratory distress syndrome
